# A Quantum–Classical Model of Brain Dynamics

**DOI:** 10.3390/e25040592

**Published:** 2023-03-30

**Authors:** Alessandro Sergi, Antonino Messina, Carmelo M. Vicario, Gabriella Martino

**Affiliations:** 1 Dipartimento di Scienze Matematiche e Informatiche, Scienze Fisiche e Scienze della Terra, Università degli Studi di Messina, viale F. Stagno d’Alcontres 31, 98166 Messina, Italy; 2 Institute of Systems Science, Durban University of Technology, P.O. Box 1334, Durban 4000, South Africa; 3 Dipartimento di Matematica ed Informatica, Università degli Studi di Palermo, Via Archirafi 34, 90123 Palermo, Italy; 4 Dipartimento di Scienze Cognitive, Psicologiche, Pedagogiche e degli Studi Culturali, Università degli Studi di Messina, Via Concezione 6, 98121 Messina, Italy; 5 Dipartimento di Medicina e Clinica Sperimentale, Università degli Studi di Messina, A.O.U. “G. Martino”, Via Consolare Valeria, 98125 Messina, Italy

**Keywords:** quantum–classical dynamics, quantum brain, open quantum systems, neuroscience, electromagnetic brain stimulation, clinical psychology, 03.65.-w, 05.30.-d, 67.10.Fj, 67.40.Db, 67.90.+z, 01.90.+g, 87.10.+e, 87.15.A-, 87.18.Bb, 87.19.La, 87.61.Bj, 87.90.+y, 87.61.Tg, 01-00, 81-03, 92C05, 92C20

## Abstract

The study of the human psyche has elucidated a bipartite structure of logic reflecting the quantum–classical nature of the world. Accordingly, we posited an approach toward studying the brain by means of the quantum–classical dynamics of a mixed Weyl symbol. The mixed Weyl symbol can be used to describe brain processes at the microscopic level and, when averaged over an appropriate ensemble, can provide a link to the results of measurements made at the meso and macro scale. Within this approach, quantum variables (such as, for example, nuclear and electron spins, dipole momenta of particles or molecules, tunneling degrees of freedom, and so on) can be represented by spinors, whereas the electromagnetic fields and phonon modes can be treated either classically or semi-classically in phase space by also considering quantum zero-point fluctuations. Quantum zero-point effects can be incorporated into numerical simulations by controlling the temperature of each field mode via coupling to a dedicated Nosé–Hoover chain thermostat. The temperature of each thermostat was chosen in order to reproduce quantum statistics in the canonical ensemble. In this first paper, we introduce a general quantum–classical Hamiltonian model that can be tailored to study physical processes at the interface between the quantum and the classical world in the brain. While the approach is discussed in detail, numerical calculations are not reported in the present paper, but they are planned for future work. Our theory of brain dynamics subsumes some compatible aspects of three well-known quantum approaches to brain dynamics, namely the electromagnetic field theory approach, the orchestrated objective reduction theory, and the dissipative quantum model of the brain. All three models are reviewed.

## 1. Introduction

The human brain is perhaps the most complicated known condensed matter system. It contains approximately $10^{2}$ billions of neurons and at least as many glia cells [1]. The brain is composed of 77 to 78% water, 10 to 12% lipids, 8% proteins, 2% soluble organic substances, and 1% carbohydrates and inorganic salts [2]. It is also extremely fascinating that higher brain functions precisely define what it means to be human. Brain states and their dynamics have so far eluded physical understanding based on molecular models. This means that one cannot describe brain dynamics by brute force, i.e., starting from the behavior of all atoms and deriving macroscopic time evolution. The problem does not only reside in the sheer number of microscopic constituents of the brain. Some of the most complex brain functions are delocalized over long distances and require synchronization processes that do not seem easy to explain only by means of the classical mechanics of atoms and molecules. In particular, the wholeness of perception requires integrating the activity of an enormous number of brain cells. Ultimately, we would like to develop a theory of brain processes where mesoscopic models can be constructed from atomistic dynamics by means of controlled approximations. With respect to this, quantum models [3,4,5,6,7,8,9,10,11,12,13,14,15,16,17,18,19,20,21,22,23,24,25,26,27,28,29,30,31,32,33,34,35] may hold the key to a possible microscopic understanding of some brain functions.

We have found that some psychological theories are based on a bi-partite logic [36,37,38,39,40,41,42,43,44,45,46,47,48] that is very similar to the logic of quantum–classical mechanics. We do wish to make clear at the very beginning that, in this paper, the words “psyche”, “psychological”, and the like are not used to address any metaphysical level of ‘reality’. While we acknowledge that such concepts lack, at the moment, both quantitative definitions and complete explanations in terms of biomolecular processes, it must be emphasized that neuroscience [49] is continuously advancing toward the inclusion of psychological phenomena within the boundaries of quantitative science. Thus, once our use of these words is understood, it becomes easier to accept the idea that psychological theories and clinical psychology could feed the synergistic growing of translational neuroscience [50,51,52,53,54,55,56], quantum models of decision-making [57,58,59,60,61,62,63,64,65], and quantum information biology [66].

In recent years, three main quantum models of the brain have been introduced in the literature. These are the electromagnetic field (EMF) approach [3,4,5,6,7,8,9,10,11], the orchestrated objective reduction (Orch OR) theory [12,13,14,15,16,17,18,19,20,21,22,23,24,25,26], and the dissipative quantum model of brain (DQMB) [27,28,29,30,31,32,33,34,35]. Even if there are several key differences between the EMF, the Orch OR approaches, and DQMB, these three theories study the brain from the perspective of condensed matter physics and matter–EMF interactions. While DQMB [27,28,29,30,31,32,33,34,35] is mainly concerned with the explanation of memory storage and retrieval, long-range correlations between brain clusters of cells and brain correlates of perception, both the EMF [3,4,5,6,7,8,9,10,11] and Orch OR [12,13,14,15,16,17,18,19,20,21,22,23,24,25,26] models were originally introduced for explaining consciousness. With regard to this, we want to state very clearly that our theory does not aim in any way to explain consciousness. Instead, we stress that our target is only to study brain dynamics in terms of physical processes. In practice, we only consider EMF [3,4,5,6,7,8,9,10,11] and Orch OR [12,13,14,15,16,17,18,19,20,21,22,23,24,25,26] insofar that they can be used as microscopic theories of physical processes in the brain.

Motivated by the germinal considerations in Refs. [3,4,5,6,7,8,9,10,11,12,13,14,15,16,17,18,19,20,21,22,23,24,25,26,27,28,29,30,31,32,33,34,35] and by the idea of a bipartite structure of logic [36,37,38,39,40,41,42,43,44,45,46,47,48], in this paper, we introduce an explicit quantum–classical model of brain dynamics. Such a model is based on the hybrid quantum–classical (QC) formalism of Refs. [67,68,69,70,71,72,73,74,75,76,77,78,79,80,81,82,83,84,85,86,87,88]. In many QC theories, the nature of the interaction between the classical and quantum subsystems is somewhat unclear and the quantum variables are not treated on the same footing as the classical DOF. The formulation of Refs. [67,68,69,70,71,72,73,74,75,76,77,78,79,80,81,82,83,84,85,86,87,88] is based on mixed Weyl symbols and is conceptually free from these drawbacks. In fact, such an approach is founded upon a statistical operator depending parametrically on phase space points. This implies that the dynamics must be considered at each phase space point without the possibility of separating quantum dynamics from the classical-like dynamics of the phase space. QC spin–boson models [89,90,91,92], and their non-linear extension [88], are appropriate for describing a finite number of quantum variables coupled to a classical DOF. Non-Hamiltonian deterministic thermostats [79,80,81,93,94,95] can be used to formulate the dissipative dynamics of mixed Weyl symbols under constant temperature conditions. The QC formalism simplifies numerical calculations of averages and response functions. In turn, response functions can be compared to electromagnetic signals that could be provided by macroscopic experiments on the brain [11,96,97,98,99,100,101,102,103,104,105,106,107,108,109,110,111,112].

We are interested in studying those brain processes that can be described in terms of a few quantum variables embedded in a classical environment. Small numbers of quantum particles are naturally found in small biological structures [20,21], and from such a scale until that of atomic nuclei [22,23,24]. Even a small number of quantum variables can have a significant effect on the dynamics of large classical systems by means of four mechanisms. One is given by non-adiabatic transitions between energy states [82,83,84,85,86,87,88]. The second one is caused by the stochastic collapse of the wave function [113,114]. The third one is generated by the motion of quantum sources of the electromagnetic fields in the brain. The fourth one is the famous ‘order from order’ mechanism elaborated on by Schrödinger [115], which led to the discovery of DNA [116,117]. All of these mechanisms are in agreement with Pascual Jordan’s idea [118,119,120] about the necessary role of the amplification of quantum processes in order to steer classical dynamics in biological environments. Hence, one can consider that single quantum particles, such as electrons and protons, retain quantum properties [82,83,84,85,86,87,88] at every temperature [121]. For such a reason, our QC approach can cope ‘almost by design’ with the controversial issue of decoherence [20,122,123,124] in warm and wet environments, such as those found in biological systems. It has also been proposed that some kind of quantum computation [125,126] might take place in the brain [20,22,23,25,26]. There are a few proposals regarding quantum computational schemes performed by means of mixed states [127,128,129,130]; however, the mainstream concept of quantum computation requires entangled states [125,126]. Entanglement is fragile and, for quantum computation to be realized at biological conditions, decoherence [122,123] should not destroy the phase coherence of quantum states at a high temperature [124]. Such a possibility is still a matter of debate [20]. For such a reason, in this paper, we do not take a quantum informational perspective [125,126].

The paper is structured as follows. We present the historic evolution of logic’s bipartite structure by discussing general semantics (GS) in Section 2.1, synchronicity in Section 2.2, and Blanco’s bi-logic in Section 2.3. In Section 3, we discuss a set of ideas in favor of quantum mechanical effects in the brain. We review the EMF approach in Section 4, Orch OR in Section 5, and DQMB in Section 6. Our QC approach is presented in Section 7. Finally, our conclusions are given in Section 8.

## 2. The Bipartite Structure of Psychology as the Root for Quantum–Classical Models of the Brain

The knowledge that some theories of the human psyche suggest that logic has a bi-partite structure [36,37,38,39,40,41,42,43,44,45,46,47,48], paralleling that of the quantum–classical world, is, for us, one of the inspiring motivations to take the first steps toward the elaboration of a quantum–classical model of brain dynamics. As is clarified in the following, bi-logic comprises Aristotelian and non-Aristotelian logics. Discussions on the bi-partite structure of logic can also be of interest to quantum models of decision making [57,58,59,60,61,62,63,64,65], quantum information biology [66], and translational neuroscience [50,51,52,53,54,55,56].

In the field of clinical psychology, Korzybski was one of the pioneers making use of non-Aristotelian logic [36,37,38,39] for therapeutical applications. A more abstract and somewhat implicit approach to such a bi-partite structure of logic can be found in the work of Jung and Pauli [40,41,42,43,44]. Instead, the most complete formulation of bi-logic and its application to clinical psychology (until now) is found in the work of Blanco [45,46,47,48], where it is called bi-logic. Since, in QM, the law of the excluded middle is not valid, so that, as in the famous Schrödinger’s example [131], a cat can be both alive and dead [125,126], at a fundamental level, quantum logic is non-Aristotelian, and we propose to identify it with Blanco’s bi-logic. If the quantum world does not follow Aristotelian logic, given that QM cannot indeed be separated from the classical world, because of the processes of ‘measurement’ and the stochastic collapse of the wave function [113,114], then physical processes must have a hybrid structure, where both Aristotelian and non-Aristotelian logic must be employed. Thus, we sustain that quantum physical processes can be described by hybrid quantum–classical models. Since bi-logic [45,46,47,48] can be likened to a quantum–classical worldview and there are already models supporting the quantum–classical nature of the brain [3,4,5,6,7,8,9,10,11,12,13,14,15,16,17,18,19,20,21,22,23,24,25,26,27,28,29,30,31,32,33,34,35], the idea of developing models by means of an explicit quantum–classical theory naturally arises. Our parallelism between bi-logic [45,46,47,48] and quantum–classical phenomena in the brain can also be considered as the motivation for extending the current quantum-like models of cognition [57,58,59,60,61,62,63,64,65] to take into account quantum–classical processes. In the remaining part of this section, we discuss the historical development of non-Aristotelian logic in psychology and clinical psychology.

### 2.1. General Semantics

Roughly speaking, GS is a specific instance of clinical psychology with the specific goal of improving mental health and adaptation to the world [36,37]. One key aspect of this approach is the claim that non-Aristotelian logic conforms more to reality. Once non-Aristotelian logic is accepted as the correct way of thinking, our language must be adjusted accordingly. GS was created with the logical structure of QM as a template [36]. An interesting connection of GS to quantum models of decision making, which is yet to be fully explored, may be founded on the free energy principle [132,133]. However, a first application of this principle to quantum decision making can be found in Ref. [57].

Although it constitutes the historical roots of many systems of clinical psychology, GS is rarely acknowledged [39]. The premises of GS are “A map is not the territory”, “A map does not represent all of a territory”, and “A map is self-reflexive”, meaning that an ’ideal’ map would include a map of the map, etc., indefinitely” [38]. These assumptions can be translated to daily life in order to improve the mental sanity of human beings [37]. In this case, GS premises become “A word is not what it represents”, “A word does not represent all of the facts”, and “Language is self-reflexive” in the sense that, in language, we can speak about language. Alas, human being reactions to verbal communication are largely based on unconscious beliefs, violating the first two assumptions and disregarding the third. Mathematics and GS are the only languages that rigorously take into account the above non-Aristotelian premises at all times. For such a reason, Korbizski strongly suggested to psychologists to study mathematical structures. On page 280 of his *Science and Sanity* [36], we find a discussion of the importance of the theory of aggregates and the theory of groups in psychology, something that will be further examined in Blanco’s bi-logic [45,46,47,48].

At variance with the general case [39], there are some instances in which the influence of Korzybski’s GS on various approaches is properly acknowledged. For example, Ellis acknowledges Korzybski’s influence on his rational emotive behavior therapy [134]. Almost similarly, Wysong pays the dues of Gestalt therapy to GS by writing a commentary in *The Gestalt Journal* [135]. How much Gestalt therapy owes to GS is also discussed in the thesis of Allen Richard Barlow [136], which is downloadable from The University of Wollongong Thesis Collection online. One of many counter-examples [39] is given by family therapy [137,138], where it is stressed that one must be aware of abstractions leading to disregarding the wholeness of processes [137] (non-elementalism [36,37]), and the difference between the verbal and the non-verbal [138] is also underlined, but without citing GS. Hence, GS may be considered (either directly or indirectly) as the hidden root of various therapeutic practices.

Given the above discussion, it is not difficult to see the logical connections between GS and QM. If we consider that scientific theories are “maps” of reality, with classical theories providing a first level of abstraction, QM is clearly characterized by a second level of abstraction. QM does not provide laws for the dynamics of models of phenomena. QM gives laws for the probability amplitudes that models of phenomena have a certain dynamics [139,140], i.e., QM provides laws for models of models. GS classifies this as the self-reflexiveness of the language. From this perspective, we can consider GS as an application of certain QM concepts to clinical psychology.

### 2.2. Pauli and Jung’s Synchronicity

The goal of the collaboration between Jung and Pauli was to find a unified view of reality in terms of both the psychological and physical point of view. Jung’s approach to the psyche was based on certain in-forming (in the sense of having the power of giving “form”) structures that he called archetypes [141]. As universal regulators of the psyche, archetypes transcended the individual and belonged to a collective unconscious, common to all humankind. From the point of view of physics, this can be deemed much less mysterious than how it sounds. Human ideas are formulated by brains that share a common physical structure. Although it has a great flexibility, such a structure may be expected to constrain the type of ideas that can be formulated. In other words, any idea that can be potentially formulated (which will be called “archetype”) must belong to the set of all ideas permitted by the common brain structure of humans. If we now call such a set “collective unconscious”, we might give a biological justification to Jung’s theory [142].

Pauli was one of the founders of QM. He interpreted QM in terms of the concept of statistical causality. This facilitated the collaboration with Jung. He explained to Jung that QM is about ‘forms’, e.g., wave amplitudes, and it is also intrinsically probabilistic. While the causality of the classical world requires the exchange of physical quantities (such as energy, momentum, angular momentum, and so on), statistical causality describes a new type of non-local correlation between systems. Such a new type of correlation is typically quantum in nature and is based on synchronistic events, i.e., random non-local coincidences [40,41,42,43,44]. One example is given by the absorption of a quantum of energy from light. The energy of the quantum is proportional to the frequency ν of the radiation and is spread out along the whole wavefront. However, because of quantum mechanical fluctuations, the energy hν, where *h* is Planck’s constant, can instantaneously disappear from the wavefront and be instantaneously transferred to a particle with resonant de Broglie frequency ν=p/2mh, where *p* is the momentum of the particle and *m* is its mass [139,140]. The time at which the quantum of energy hν disappears from the wavefront and instantaneously reappears as belonging to the particle’s kinetic energy (synchronistic events) is completely random. It seems a pure ‘coincidence’. In practice, the formalism of creation and destruction operators of quantum field theory [143,144] describes quantum processes in terms of random disappearances and reappearances of energy quanta to and from different systems. However, everything is probabilistic. There is no reason for something to happen at a given time or for the quanta to reappear in the energy of one particle instead of another one. The word ‘coincidence’ can indeed be considered appropriate.

Analogously, Jung considered random coincidences in the classical world as the analogue of the statistical causality in the quantum world. Moreover, in Jung’s theory, random coincidences were also the origin of subjective meaning. This also means that the organizing principle of reality, which Jung called synchronicity, is found in meaningful coincidences. Afterwards, the concept of synchronicity was further generalized to include acausal correlations without any psychological component. We can conclude that Jung’s synchronicity reflects the quantum–classical nature of the world.

### 2.3. The Bi-Logical Structure of Psychology

Korzybiski’s GS [36] proposes a new psychology founded on mathematical structures and, to this end, briefly dealt with both set and group theory. However, it is only in the work of Blanco [45,46,47,48] that these ideas are fully exploited in order to generalize Freud’s formulation of the unconscious. Whereas Freud defined the unconscious in a qualitative way, i.e., what is hidden and repressed in the psyche, Blanco describes it as a bipartite structure. Such a bipartite structure has one side that is asymmetric (which we may call Aristotelian by following GS language), pertaining man’s common-day experience, and another side that is symmetric (which we may call non-Aristotelian), where space and time do not exist and the logical principle of non-contradiction is no longer valid. Blanco stated that both logics are at work in the human psyche [45,46,47,48] and that clinical practice must accurately take into account this point.

Blanco’s and GS’s conceptual structures share concepts taken from QM. However, whereas GS is fully non-Aristotelian (without any form of classical-like logic attached to it), Blanco’s bi-logic has an Aristotelian component (congruent with a classical worldview) and another non-Aristotelian component (in agreement with the logic of QM). Taking both aspects into account, we conclude that Blanco’s bi-logic [45,46,47,48] formulates a QC conceptual perspective of the psyche, reflecting the QC nature of the phenomenological world. A full acknowledgement of this parallelism and its possible consequences on clinical practice are a matter of novel research.

## 3. Schrödinger’s ‘Order from Order’ and Jordan’s Quantum Amplification

In this section, we want to put into evidence two quantum mechanical effects that we consider fundamental for biological matter [120,121] in general and for the brain in particular. Our quantum–classical model can be designed so that it manifestly incorporates them. One such effect is Schrödinger’s ‘order from order’ [115], and the other is Pascual Jordan’s quantum amplification [118,119,120].

Classical mechanics applied to biological matter, including the brain, exploits statistical fluctuations, i.e., the mechanism that Schrödinger called ’order from disorder’ [115]. What Schrödinger actually wanted to express with the expression ’order from disorder’ is that there are some ordered macroscopic structures that can arise from the statistical disorder at the microscopic level. In truth, ‘microscopic statistical disorder’ is a misnomer that stands for the great number of microscopic states that correspond to the same macroscopic state [145,146]. Von Neumann entropy (and its quantum–classical generalization defined in terms of the mixed Weyl of the statistical operator) is a property of the macrostates given in terms of the probability of microstates [147,148]. The belief that the passage to macroscopic ‘order’ is associated with an entropy decrease is mistaken. The first reason is that macroscopic ‘order’ is somewhat an anthropomorphic concept that can only be defined once some macroscopic variables are chosen. On the contrary, microscopic order is physical since it is defined in terms of the number of microstates that are compatible with the macroscopic constraints. A system must be considered microscopically ordered if there is a small number of states associated to the macroscopic constraints. In agreement with the third law of thermodynamics, for example, this takes place at T=0, where there is only one accessible microstate and the system is maximally ordered on the microscopic level. Another example is given by the phenomenon of re-entrant phase transitions [149,150,151,152], where the macroscopic ‘ordered’ phase has a higher entropy than the microscopic ‘disordered’ one because of the unfreezing of certain DOF. Irreversible microscopic dynamics, such as diffusive motion, does not conserve the number of accessible microstates of the system conditioned by the macroscopic constraints and thus leads to an increase in entropy [147,148]. This is the essence of Schrödinger’s ’order from disorder’ mechanism [115]: in our macrocosm, we are surrounded by structures that we classify as ordered but that are based on microscopic disorder in agreement with the second law of thermodynamics.

As discussed by Schrödinger, an ’order from disorder’ mechanism can explain neither the stability of biological information nor the synchronization of molecular processes. To explain living matter, Schrödinger proposed a second mechanism that he named ’order from order’. The mechanism of ’order from order’ is basically founded on a quantum-mechanical zero-temperature clockwork in agreement with the third law of thermodynamics [115]. Only solid forms of matter allow for quantum clockworks to exist in a high-temperature disordered biological environment. This is caused by the existence of energy gaps protecting, e.g., long-wavelength electronic wavefunctions in solids. This idea led Schrödinger to predict that an aperiodic solid (ultimately identified with DNA [116,117]) would contain, in a stable manner, the information needed by the living organism to survive entropic decay. Currently, the idea has become more general and is not limited to solid structures as shields from molecular disorder. One example is found in the Orch OR theory, according to which quantum effects are protected inside hydrophobic regions of biological microstructures [20,21]. Another mechanism used to protect the quantum clockwork is provided by rigid boundaries enclosing quantum variables [153,154,155].

Pascual Jordan’s idea [118,119,120] about how quantum mechanics can steer the dynamics of a classical biological environment also works for systems with no genetic code and, as such, is more general than the ‘order from order’ mechanism. Jordan introduced the concept of quantum amplification. Basically, this consists of interpreting the stochastic collapses [113,114] of a quantum state as a way to funnel information from a smaller-scale level to a classical higher-scale level, i.e., an amplification. Even a small number of quantum variables can produce a quantum amplification through the collapse of their state, and can have a significant effect on the dynamics of large classical systems. Quantum amplification is also at work in non-adiabatic transitions between energy states of a quantum subsystem [82,83,84,85,86,87,88] through the back-reaction onto a classical environment, which the quantum subsystem interacts with.

Both the mechanisms of ‘order from order’ and quantum amplification are in some way present in the quantum approaches to brain dynamics [3,4,5,6,7,8,9,10,11,12,13,14,15,16,17,18,19,20,21,22,23,24,25,26,27,28,29,30,31,32,33,34,35] that we are going to discuss in the following. However, since we are explicitly identifying them, we will be able to design our quantum–classical model in order to take them into account in a general way.

In addition to the mechanisms of ‘order from order’ [115] and quantum amplification [118,119,120], currently, quantum informational approaches [125,126] are routinely invoked to understand, e.g., condensed matter [156] systems. However, we believe that the idea of considering a biological systems akin to a quantum computing machine is somewhat controversial. As we try to explain in this paper, it is even more controversial than quantum biological theory based on the properties of a few quantum particles, on the ’order from order’ mechanism [115], and on the quantum amplification process [118,119,120]. The reason for this is that, despite a few suggestions [127,128,129,130], entanglement is considered to be a necessary resource for quantum computers [125,126]. However, the main resource of entanglement is the persistent quantum coherence that, because of thermal disorder and decoherence, cannot commonly last for time intervals long enough to perform robust quantum computations inside the brain. Nonetheless, it has been proposed to interpret brain processes as a form of quantum computing [20,22,23,25,26]. It is still a matter of debate that such a type of computation can take place in the brain [20] nothwithstanding decoherence [122,123,124].

Hence, we think that quantum processes in the brain can be studied from the perspective of quantum biology [3,120] with a somewhat less controversial approach. Henceforth, we will not pursue the quantum informational perspective [125,126].

## 4. Electromagnetic Fields in the Brain

In this section, we discuss the EMF field approach to brain dynamics [3,4,5,6,7,8,9] and propose its generalization to include those quantum effects that can be influenced by the various forms of electromagnetic brain stimulation [96,97,98,99,100,101,102,103,104] and that can be observed by EEG [8,99,112].

The role of EMFs in bridging space and time scales is very important [3,4,5,6,7,8,9,10,11]. Brain states are routinely studied via computer simulation [157] and various noninvasive stimulation techniques, such as alternating current stimulation (ACS) [96,97,98,99,100] and transcranial direct-current stimulation (tDCS) [101,102]. In particular, tDCS is one of the most investigated methods in the field of non-invasive brain stimulation. It modulates the excitability of the cerebral cortex with direct electrical currents (1 ≈ 2 mA [103]) delivered via two or more electrodes of opposite polarities (i.e., anode and cathode) placed on the scalp. tDCS modulates resting neuronal membrane potentials at sub-threshold levels [101], with anodal and cathodal stimulation increasing and decreasing cortical excitability, respectively [102]. Although their tDCS-induced physiological mechanisms are not yet fully understood, it is assumed that effects are based on long-term potentiation (LTP) and long-term depression-like (LTD) mechanisms [102,104].

In the history of brain research, it was assumed that higher brain functions, such as learning and memory, arise from electrical impulses passing through neurons. The physical explanation of permanent information storing was assigned to multiple reflections of impulses through neuronal circuits [158,159]. This idea is basically exemplified by the Hodgkin–Huxley model [160,161]. Despite its undoubted success, some limitations of this model have been discussed [162,163,164,165,166] and possible generalizations have been suggested [167,168,169]. From our perspective, the discussion regarding the role of ion channels [168,169] is particularly important. The relevance of quantum effects for charge transport in ion channels has been strongly supported in Refs. [170,171]. Typically, this implies that ion channels’ selectivity may be founded on quantum dynamics [172,173,174]. Ultimately, this line of research impinges on possible extensions of the Hodgkin–Huxley model, not only to take into account the description of ion channels’ conductance but also to incorporate quantum effects [175].

The idea that other physical agents, rather that the sole dynamics of neural networks, must be invoked to describe highly coordinated brain activity is not new [11,105,106,107]. Electric charges (e.g., electrons, protons, ions), together with their associated currents, are the sources of EMFs [3,4,5,6,7,8,9,10]. In turn, these EMFs interact with water dipoles and also influence van der Waals and Casimir interactions among brain macromolecules. ACS has shown the importance of EMFs in the brain [96,97,98,99,100], and tDCS of human subjects [101,102] has shown the importance of both cognition processes and psychological state changes, which can be modulated. For instance, anodal (excitatory) tDCS of the prefrontal cortex boosts affective memory, such as fear extinction learning [108,109,110]. Moreover, the cathodal (i.e., inhibitory) stimulation of the tongue motor neurons of the primary motor cortex reduces appetite [111].

The working of tDCS might be understood through a mechanical analogy. The complex dynamics of brain EMFs can be reduced to the time evolution of their sources. Such dynamics can be mapped onto that of a harmonic spring mattress. Within this pictorial description, tDCS can be equated to the nonlinear effect generated by the application of a constant pressure to specific extended regions of the spring mattress. The applied pressure changes the harmonic dynamics of the mattress so that oscillations with principal frequencies (phonons) scatter with each other. This mechanical model might be useful for performing computer simulations of certain processes that are observed in tDCS. We note that the same model has been used to give a pictorial representation of quantum fields [143]. Both ACS and tDCS provide evidence that brain EMFs are not ephemeral; they are correlated to the dynamics of their sources, but also react back and influence both cognitive functions and emotions.

When studying brain dynamics on the mesoscopic scale of EMFs, it may seem that there is no necessity to invoke any quantum effect. The original EMF approach was formulated only in terms of classical physics [3,4,5,6,7,8,9,10,11]. Nevertheless, our analysis below can elucidate the fundamental quantum coherent properties of the microscopic EM fields invoked by such an approach. [143,144,153,154,155,176,177]. Observable coherent EMFs have, by definition, a well-defined phase. Quantum mechanically, phase Φ and photon number *N* are conjugate variables. This implies that they obey the indeterminacy relation

(1) ΔΦΔN≥1.

According to Equation (1), when the number of quanta of the photon field *N* is not fixed and ΔN can be large, it follows that the phase Φ is well determined and the quantum photon field is coherent.

The only way for the number of photons *N* to fluctuate is that photons are continuously absorbed and re-emitted. In other words, coherent EMFs are ‘composed’ of virtual photons [176], e.g., packets of energy in momentum space whose existence is ephemeral. Interestingly, experimental evidence shows that dendrimers can act as a trap for photons [153,154]. According to quantum electrodynamics [144], a trapped photon can be represented in terms of virtual photons continuously emitted and re-absorbed between fermions. This picture can be developed considering that, in terms of Feynman diagrams, a photon line connecting two fermion lines is a virtual photon describing Møller scattering [144]. Thus, an exchange of virtual photons along the time direction between the two fermion lines, generating a so-called “ladder” diagram [178,179], may very well be considered the microscopic picture of a trapped photon.

## 5. Penrose and Hameroff’s Orch OR

Orch OR theory [12,13,14,15,16,17,18,19,20,21,22,23,24,25,26] provides a detailed molecular mechanism for the time evolution of brain states. According to Orch OR theory [12,13,14,15,16,17,18,19,20,21,22,23,24,25,26], quantum effects in tubulin proteins (which are organized in arrays of microtubules inside the cytoplasm of brain cells) play an important role in brain function. Quantum dynamics of the electronic orbitals of carbon rings inside tubulins, time evolution of the nuclear spins, quantum energy transport among microtubules, and the spontaneous collapse of microtubules’ wave function are the main ingredients of this theory. Upon collapse of the wave function, classical brain dynamics ensues. For example, we look at charges’ and masses’ tunneling as an event arising from the collapse of these variables’ wavefunctions. In turn, such a collapse induces the collapse of the environment’s and EMF’s quantum states, triggering chemical reactions, diffusion processes, macroscopic currents, and so on.

One peculiar characteristic of Orch OR is that neurons are not considered to be the fundamental units of information processing [11]. Instead, in Orch OR, it is proposed that information processing takes place in ordered arrays of microtubules inside the cell. This idea slowly took form during the 1980s and the first part of the 1990s when Hameroff noticed the effects of anesthetics on networks of microtubules inside the cell. In a series of papers, Hameroff et al. [180,181,182,183,184,185] proposed that some kind of digital computation was taking place in arrays of microtubules. Such a computation was based on nonlinear electrodynamic effects [180,181,182,183,184,185]. However, the question of how the results of local digital calculations could be efficiently transferred between distant brain regions by classical diffusive mechanisms remained. Hence, Hameroff started his search for different mechanisms. On a different path, looking for a fundamental explanation of wave function collapse in QM, Penrose elaborated the theory of objective reduction (OR) [12,13,14,15,16].

In the standard interpretation of QM, the collapse of the wave function, i.e., the transition from the world of possibilities to that of classical events [186,187], is explained only through the stochastic interaction of quantum systems with classical ones. The collapse of the wave function is called the ‘measurement’ process because of the interaction with a classical system [188]. It is not explained within the theory but it is assumed as a postulate. OR proposes that the superposition of different stationary mass distributions becomes unstable because of quantum gravitational effects, and, beyond a certain time interval threshold, it naturally collapses according to the standard probabilistic rules of QM, but without any external intervention of a “measuring instrument”. A simple way to discuss this process is to consider

(2) $$\omega_{\mathrm{Bohr}}=\Delta E/\hbar \;,$$

as the Bohr frequency of the energy eigenvalues of two eigenstates involved in a certain superposition. Penrose gives a number of reasons for why the superposition must become unstable in the presence of quantum gravitational effects. The lifetime of the superposition is given by

(3) $$\tau \approx \frac{h}{\Delta E}\;.$$

Looking at Equations (2) and (3), one might say that, in a certain sense, the deterministic time evolution of the gravitational field acts as the instrument measuring the superposition. However, according to Penrose [12,13,14,15,16], there is an important difference between the measurement of the superposition by a classical instrument and by a quantum gravitational field. A measurement performed by a quantum gravitational field is still a fully quantum mechanical process and, as such, is intrinsically random and absolutely non-computable. Penrose considered that brain dynamics is interspersed with discrete events (see Ref. [7] for experimental support of this idea). On a phenomenological basis, such events parallel the discontinuity of wakefulness and awareness [7] and other rhythmic phenomena in the brain. Penrose identified discrete events in the brain with a series of wave function collapses. Between one collapse and the other, the brain can evolve coherently so that new superpositions are formed. We note that such a coherent evolution of the wave function, interrupted by quantum gravitational collapses, is reminiscent of both piecewise deterministic processes in open quantum systems [189] and nonadiabatic dynamics of the QC system on an adiabatic basis [85,86,87,88].

While Penrose put forth the idea that OR could have an important role in brain dynamics, Hameroff fleshed out the detailed biomolecular mechanisms. Inside each tubulin protein making up a given microtubule, Hameroff hypothesized the existence of quantum matter systems able to support stable quantum dynamics in between OR events. One example is given by carbon rings and their delocalized molecular orbitals, which can evolve coherently in a superposition of states. The carbon rings are pushed by hydrophobic forces into the tubulin’s interior, shielding them from the decoherence [122,123] caused by the polar environment outside the protein. The carbon rings form helical structures inside each microtubule. They also create oriented arrangements that can act as quantum channels [20,21] through which quantum signals travel among the lattice of microtubules inside the cell’s cytoskeleton.

Various types of quantum oscillators are therefore found in microtubules’ ordered structures, e.g., time-dependent electric fields arising from the dynamic polarization of molecular charges (which produce van der Waal and Casimir–Polder forces), magnetic fields originating from electron spin dynamics, etc. Notably, it has also been suggested [22,23,24] that nuclear spins can play an important role in Orch OR theory since they are shielded from decoherence for longer time intervals than other quantum systems in the brain. Recently, this theory [22,23,24] has gained experimental support [25]. The frequencies of all such quantum oscillators range from kilohertz to terahertz. Orch OR theory requires the feedback [26] between the quantum coherent evolution of microtubules and, for example, the classical dynamics of microtubule-associated proteins (MAPs) [190,191]. Such classical dynamics concerns the classical evolution of MAPs [190,191] and CAMKII [192,193,194,195,196,197], viz.the direction of motion, the place where MAPs and CAMKII halt their motion, the case in which they interact or do not interact with the tubulins, and the precise time when they interact. According to the Orch OR theory [12,13,14,15,16,17,18,19,20,21,22,23,24,25,26], the coherent evolution of the microtubule’s wave function and its OR determine all detailed molecular events. However, we must note that the possibility that extended brain regions may be free from decoherence [20,122,123,124] is rather controversial.

Lately, there has been a convergence of ideas between the approach to brain dynamics via quantum EMFs [153,154,176] and Orch OR [198]. The physical process underlying quantum signaling in Orch OR has been assumed to be photon emission. Due to the work of Alexander Gurwitsch, it has been known since the beginning of the 20th century that tissues inside the body emit biophotons [199,200,201]. Such biophotons may be supported by the hydrophobic interior region of tubulins, where tryptophanes, with their indole rings of π electron orbitals forming optically active molecular orbitals, are found. The packing of indole rings may give rise to resonant energy transfer between molecular orbitals [198] much in the same way that Förster resonant energy transfer takes place between close chromophores. Kurian et al. [202] represented the microtubule as a chain of two-level systems and calculated the coupling constants in the Hamiltonian by means of molecular dynamics simulations and quantum chemical calculations. Exciton propagation was performed by means of the Haken and Strobl method [203]. Their main result is that energy transfer occurs on a length scale of at least microns. What is even more interesting from the quantum optical perspective is that Kurian et al.’s simulation [202] does not consider the geometric structure of the left-handed helixes of microtubule in mammals. There are reasons to believe that super-radiance can be important in such complicated geometric arrangements [204,205,206]. Very recently, the experimental study of Kalra et al. [207] found that photonic energy transfer in microtubules occurs over 6.6 nm, cannot be explained in terms of Föster theory, and is damped by anesthetics. The idea that electromagnetic resonance is the fundamental mechanism of communications among molecules was first proposed by Veljkovic et al., who also suggested that such a mechanism could provide long-range effective communication [208]. At this stage, we believe that a unification of the EMF and Orch OR theories of brain dynamics is conceptually very probable [177].

Nevertheless, the Orch OR model remains very controversial. It is based on quantum gravitational effects used to objectively induce the wave function collapse by using only a provisional theory of quantum gravity [17,18,19].

## 6. The Dissipative Quantum Model of Brain

The precursor of DQMB [27] was the seminal paper [209] of Ricciardi and Umezawa, where the quantum field theory model of brain (QFTMB) was introduced [210,211]. An interacting QFT can naturally describe the creations of dynamical correlations. Whenever a quantum field has an average value different from zero in the vacuum, the vacuum state will no longer be unique. Instead, there will be different vacua and each of them will spontaneously break the symmetry of the Hamiltonian density [212,213,214]. In order to compensate for the SSB, the proliferation of bosonic modes, establishing long-range correlations with the local configurations of the field, sets in. The symmetry-breaking mechanism in the QFTMB [209] can qualitatively describe both long-term memory storage in the ground states with broken symmetry and long-range correlations between distant clusters of neurons by means of the Nambu–Golstone bosons. Nambu–Goldstone bosons also act as the agents for memory retrieval [209] while excited energy states of the field describe short-term memory.

In DQMB, the dissipation is ascribed to excited thermal states, which are represented through doubling the number of fields according to thermo field dynamics [210,211]. DQMB also predicts that long-range correlations between distant excited areas of the brains do not occur via chemical transport but by means of Nambu–Goldstone bosons [212,213,214]. One example of such long-distance correlations is observed when the brain is locally stimulated. In this case, there is experimental evidence [96,97,98,99,100,101,102] that the response is given by simultaneous excitations in several regions [215,216], which are far from one another. In DQMB, quantum coherent fields interact with classical neurons and glia cells. DQMB presents us with a hybrid description where memory storage finds a quantum explanation and biochemical reactions find a classical one. Such a hybrid description requires to coarse-grain the classical degrees of freedom (DOFs) and to describe them in terms of some kind of waves. Only at this level of description is it possible to formulate the interaction between the Nambu–Goldstone bosons [212,213,214], the condensed quantum field predicted by the model, and the classical waves, much in the same way that phonons in an ordered solid interact with acoustic waves [27,28,29,30,31,32,33,34,35].

Due to its mesoscopic nature, DQMB does not aim at describing the behavior of the molecular constituents of the brain with atomistic detail, e.g., neurons, glia cells, membranes, neurotransmitters, or other macromolecules. Today, we know that all of these structures form brain clusters [215,216] that, once stimulated [96,97,98,99,100,101,102], can influence human behavior [99,100]. Since normal mesoscopic brain dynamics is not chaotic, the brain’s response to stimuli cannot be expected to depend on the number *N* of the fundamental constituents of the clusters. If *N* is not fixed, Equation (1) is valid, the phase Φ of the matter field is well defined, and the matter field will be coherent. Moreover, DQMB does not specify the physical nature of the bosonic fields of the brain. The bosonic fields in Fourier space may be identified with the modes of the quantum oscillators considered in Orch OR theory [20,21] and discussed in Section 5. However, another proposal suggested to interpret the bosonic fields in terms of the dipoles of water molecules [217,218,219,220,221]. According to the theory in Refs. [217,218,219], when water molecules have a high density, the approximation of weak coupling to the electromagnetic vacuum field [222] may not hold. It has been suggested that water in the cytoplasm is found in a structured state [223], so the considerations of Refs. [217,218,219] are definitely relevant for brain dynamics. Since a water molecule is dipolar, a coherent superposition of the dipoles of many water molecules can be described by a coherent quantum dipolar field. Hence, in this model, it is the condensation of the quantum dipolar field that produces a ground state with broken symmetry, i.e., many unitarily inequivalent subspaces [224]. Consequently, Nambu–Goldstone modes arise for restoring symmetry at long range.

In the following, we use the Hamiltonian of the noninteracting dipolar wave quanta of Ref. [35] in order to elucidate the theoretical description of dissipation by means of doubling the DOFs as described by Umezawa’s thermo field dynamics [210,211]. The dynamical variables of DQMB are doubled upon introducing creation and annihilation operators of physical dipolar wave quanta, ${\hat{a}}^{†},\hat{a}$, respectively, and dual creation and annihilation operators of fictitious dipolar wave quanta, ${\hat{v}}^{†},\hat{v}$, respectively. For example, the Hamiltonian of the noninteracting dipolar wave quanta might be defined as [35]:

(4) $${\hat{H}}_{0}=\sum_{k}\hbar \omega_{k}\left({\hat{a}}_{k}^{†}{\hat{a}}_{k}-{\hat{v}}^{†}{\hat{v}}_{k}\right)\;,$$

where $\omega_{k}$ is the oscillation frequency of each mode. The interaction between the physical modes and their doubles can be taken as

(5) $${\hat{H}}_{I}=i\sum_{k}\hbar \gamma_{k}\left({\hat{a}}_{k}^{†}{\hat{v}}^{†}-{\hat{a}}_{k}{\hat{v}}_{k}\right)\;,$$

where $\gamma_{k}$ is the damping constant of each mode. Finally, the total many-body Hamiltonian of the thermal system is

(6) $$\hat{H}={\hat{H}}_{0}+{\hat{H}}_{I}.$$

A thorough study of the Hamiltonian in Equation (6) and its associated equations of motion has led to finding a number of interesting results over the years [30].

DQMB has been applied by Vitiello and collaborators to study various brain processes [29]. Some applications include nonlinear dynamics [31], cortical patterns in perception [32], the relation between fractal properties and coherent states in the brain [33], rhythmic generators in the cortex [34], and correlations of brain regions that are realized through entanglement [35]. DQMB dynamics has also been adopted by Nishiyama et al. in a number of works [225,226,227,228]. As reported in Ref. [225], one notes that the phenomenon of super-radiance, which is expected to occur in complicated geometric arrangements of microtubules, also occurs in DQMB.

## 7. The Quantum–Classical Model of Brain

Our aim is to model multi-scale brain dynamics, explicitly treating classical DOFs and quantum variables on the same footing. To this end, our approach considered mixed Weyl symbols of dynamical variables (represented by operators in the standard formulation of quantum mechanics) and a mixed Weyl symbol of the statistical operator (corresponding to the density matrix of the systems in the standard representation of QM) [67,68,69,70,71,72,73,74,75,76,77,78,79,80,81,82,83,84,85,86,87,88]. In this regard, it is worth remarking that we introduced a quantum–classical model of brain dynamics because, while thermal disorder, decoherence, and a short de Broglie wavelength require treating the DOFs of massive atoms and molecules by means of classical mechanics, there are also intrinsically quantum variables at all temperatures influencing the dynamics of the classical DOF. Such quantum variables are, e.g., tunneling electrons and protons, nuclear and electron spins, electronic-state-associated phenyl rings, photons, and so on. Our working hypothesis is that certain brain processes can most naturally be understood in terms of the above picture. In the following, we sketch the quantum–classical theory that we used and introduce a general Hamiltonian model that can be adapted to perform calculations of specific phenomena at the interface between the quantum and the classical world. For example, we plan to study if quantum dynamics can explain ion channels’ selectivity inside the classical membrane of a brain cells. Quantum particles that are recognized can be characterized in terms of their vibrational spectra. Response functions can be calculated depending of the oscillators’ frequencies and the wave vectors, and these can be compared to the results of electromagnetic brain stimulation and EEG. Another example is given by the trapping of photons by neurons’ dendrites. Once trapped, photons should acquire an effective mass and the dynamics of the dendrites should be slowed down. One could expect that some observable effect of or alteration in neuronal dynamics should follow. The question is if this investigation could explain the effects of electromagnetic brain stimulation. These are just a few examples. In general, numerical simulations can be used as thought experiments for ‘discovering’ the qualitative agreement between a specific model and real experiments or the causal connection between the numerical values of the parameters of the model and the behavior of the model itself. Comparisons between different Hamiltonian models can also lead to a better understanding of the essential features of a given phenomenon. These are the goals of our quantum–classical model of brain dynamics. Section 2 has logically sustained the choice of adopting a quantum–classical approach and has pointed us toward the very ambitious goal of developing models that cannot only go from the microscopic level to the results of electromagnetic brain stimulation but that can also reach the psychological states of the brain. We intend this as specified by a few macroscopic variables that can be related to human behavior. If human behavior can be affected by electromagnetic brain stimulation and if we are successful in building models going from the quantum–classical microscopic level to brain stimulation, then our goal will be reached, even if it is as an overall result of a research activity whose first results are just those proposed in this article.

We imagine that the brain is described by quantum operators $\left(\hat{r},\hat{p},\hat{R},\hat{P}\right)$, where $\left(\hat{r},\hat{R}\right)$ are position operators and $\left(\hat{p},\hat{P}\right)$ are the respective conjugated momenta operators. Now, $\left(\hat{r},\hat{p}\right)=\hat{x}$ corresponds to the brain variables with a long de Broglie wavelength that, for this reason, must be treated quantum mechanically, whereas $\left(\hat{R},\hat{P}\right)=\hat{X}$ can be treated semi-classically because of their much shorter de Broglie wavelength. A partial Wigner transform over the $\left(\hat{X}\right)$ operators [77] introduces the mixed Weyl symbols $\tilde{O}\left(X\right)$ and $\tilde{W}\left(X\right)$ arising from $\hat{O}\left(\hat{x},\hat{X}\right)$ and $\rho (\hat{x},\hat{X})$, respectively. Please note that the following notation is adopted: when a quantum operator depends both on quantum variables and classical DOFs, $\tilde{a}$ is written on it, whereas, if the quantum operator does not depend on *X*, $\hat{a}$ is used. No hat is used in the case of a dynamical variable depending only on *X*. A practical example of a possible application of this mixed QC representation can be given when considering molecular orbitals, electron and nuclear spins, light ions, neurons, glia cells, and electromagnetic interactions. Conformational dynamics of cells may be represented through phonons, i.e., harmonic DOFs. Other harmonic DOFs can be used to describe coherent EMFs. The inclusion of non-Harmonic perturbation terms provides a description of non-trivial interactions among all of the DOFs of the model. Zero-point effects on the motion of classical-like DOFs can be described by means of advanced algorithms that will be explained in the following. As in the case of DQMB, the goal is to set up a mesoscale approach to brain dynamics, noting, however, that, in our case, the QC dynamical variables are explicitly represented.

If we now introduce the coordinates of the EMF modes (Q,Π)=Y, a possible model mixed Weyl symbol of the Hamiltonian $\tilde{H}\left(X,Y\right)$ can be written as

(7) $$\begin{array}{ccc} \tilde{H}\left(X,Y\right) & = & {\hat{H}}_{S}+H_{B}\left(X\right)+H_{F}\left(Y\right)+{\tilde{V}}_{\mathrm{SB}}\left(R\right)+{\tilde{V}}_{\mathrm{SEM}}\left(Q\right) \end{array}$$

In Equation (7), ${\hat{H}}_{S}\left(t\right)$ is the Hamiltonian operator of the quantum subsystem with quantum variables $\hat{x}$. The phononic Hamiltonian is

(8) $$H_{B}\left(X\right)=\sum_{J=1}^{N_{\mathrm{PH}}}\left(\frac{P_{J}^{2}}{2}+\frac{{\left(\omega_{J}^{\mathrm{PH}}\right)}^{2}}{2}R_{J}^{2}\right)\;,$$

where $\omega_{J}^{\mathrm{PH}}$, $J=1,...,N_{\mathrm{PH}}$ is the frequency of each phonon. Similarly, the EMF Hamiltonian is

(9) $$H_{F}\left(Y\right)=\sum_{K=1}^{N_{\mathrm{EM}}}\left(\frac{\Pi_{K}^{2}}{2}+\frac{{\left(\omega_{K}^{\mathrm{EM}}\right)}^{2}}{2}Q_{K}^{2}\right)\;,$$

where $\omega_{K}^{\mathrm{EM}}$, $K=1,...,N_{\mathrm{EM}}$ is the frequency of the EMF mode. The interaction operators ${\tilde{V}}_{\mathrm{SB}}\left(R\right)$ and ${\tilde{V}}_{\mathrm{SEM}}\left(Q\right)$ describe the coupling of the phonons and of the EMF to the quantum subsystem, respectively. Assuming, for simplicity, a bilinear approximation, these can be written as

(10) $$\begin{array}{ccc} {\tilde{V}}_{\mathrm{SB}}\left(R\right) & = & -\sum_{J=1}^{N_{\mathrm{PH}}}C_{J}R_{J}\hat{\chi } \end{array}$$

(11) $$\begin{array}{ccc} {\tilde{V}}_{\mathrm{SEM}}\left(Q\right) & = & -\sum_{K=1}^{N_{M}}F_{K}Q_{K}\hat{\zeta }\;, \end{array}$$

where the $C_{J}$ and $F_{K}$ are the coupling constants of the quantum operators $\hat{\chi }$ and $\hat{\zeta }$, respectively. The operators $\hat{\chi }$ and $\hat{\zeta }$ act on the same space of $\hat{x}$.

The dynamics of the mixed Weyl symbol $\tilde{O}\left(X,Y,t\right)$ of an arbitrary operator $\hat{O}$ is given by a QC bracket [67,68,69,70,71,72,73,74,75,76,77,78,79,80,81,82,83,84,85,86,87,88]. The QC bracket is a quasi-Lie bracket [78,79,80,81] that breaks the time-translation invariance of Lie algebras because it does not satisfy the Jacobi relation. In the case of a system with both phononic and EMF modes, it can be written by introducing two antisymmetric matrices, $\Omega =-\Omega^{-1}$ and $\Lambda =-\Lambda^{-1}$:

(12) $$\Omega =\left[\begin{array}{cc} 0 & 1\\ -1 & 0 \end{array}\right]$$

and

(13) $$\Lambda =\left[\begin{array}{cccc} 0 & 0 & 1 & 0\\ 0 & 0 & 0 & 1\\ -1 & 0 & 0 & 0\\ 0 & -1 & 0 & 0 \end{array}\right]\;.$$

The QC equation of motion in the Heisenberg picture reads

(14) $$\begin{array}{ccc} \partial_{t}\tilde{O}\left(t\right) & = & \frac{i}{\hbar }\left[\begin{array}{cc} \tilde{H} & \tilde{O}\left(t\right) \end{array}\right]\Omega \left[\begin{array}{c} \tilde{H}\\ \tilde{O}\left(t\right) \end{array}\right]-\frac{1}{2}\tilde{H}\overset{\leftarrow }{\nabla^{X,Y}}\Lambda \vec{\nabla^{X,Y}}\tilde{O}\left(t\right)\\ & + & \frac{1}{2}\tilde{O}\left(t\right)\overset{\leftarrow }{\nabla^{X,Y}}\Lambda \vec{\nabla^{X,Y}}\tilde{H}\;, \end{array}$$

where $\nabla^{X,Y}=\left(\left(\partial /\partial R\right),\left(\partial /\partial Q\right),\left(\partial /\partial P\right),\left(\partial /\partial \Pi \right)\right)$ is the phase space gradient operator.

The lhs of Equation (14) defines the quantum–classical bracket of $\tilde{O}\left(t\right)$ with $\tilde{H}$. The first term in the lhs of Equation (14) is the quantum commutator, whereas the other two terms are Poisson brackets. All terms are written in matrix form [79,80,81]. The super propagator associated to the QC bracket is

(15) $$\begin{array}{ccc} \tilde{\tilde{U}}\left(t\right) & = & \exp\left\{\left(it/\hbar \right)\left[\begin{array}{cc} \tilde{H} & \ldots \end{array}\right]\Omega \left[\begin{array}{c} \tilde{H}\\ \ldots \end{array}\right]-\left(t/2\right)\left(\tilde{H}{\overset{\leftarrow }{\nabla }}^{X,Y}\Lambda {\vec{\nabla }}^{X,Y}\ldots \right)\right.\\ & + & \left.\left(t/2\right)\left(\ldots {\overset{\leftarrow }{\nabla }}^{X,Y}\Lambda {\vec{\nabla }}^{X,Y}\tilde{H}\right)\right\} \end{array}$$

The super-operator $\tilde{\tilde{U}}\left(t\right)$ defines the dynamics of mixed Weyl symbols of standard operators as

(16) $$\tilde{O}\left(t\right)=\tilde{\tilde{U}}\left(t\right)\tilde{O}\;,$$

where $\tilde{O}=\tilde{O}\left(t=0\right)$. QC averages are calculated using the formula

(17) $$\langle \tilde{O}\left(t\right)\rangle ={\mathrm{Tr}}^{\prime }\int dXdY\;\tilde{W}\left(X,Y;t\right)\tilde{O}\left(X,Y,t\right)\;.$$

In Equation (17), the parametric time dependence of the mixed Weyl symbol of the statistical operator of the system, $\tilde{W}\left(X,Y;t\right)$, describes possible non-equilibrium initial conditions. The formalism presented here can be easily adapted to more general non-equilibrium situations arising from an explicit time dependence of the mixed Weyl symbol of the Hamiltonian in Equation (7). In such a case, it would be more convenient to adopt the Schrödinger scheme of motion and propagate the mixed Weyl symbol of the statistical operator. One would also have to take into account the time ordering of the propagator, something that can be implemented by the algorithm [86]. Non-equilibrium dynamics is important if one considers the free energy principle proposed by Karl Friston [132,133]. Recently, such a direction of research has witnessed interesting developments [57]. As for QC correlation functions, they are defined in the following way:

(18) $$\langle {\tilde{O}}_{1}\left(t\right){\tilde{O}}_{2}\rangle ={\mathrm{Tr}}^{\prime }\int dXdY\;\tilde{W}\left(X,Y;t\right){\tilde{O}}_{1}\left(X,Y,t\right){\tilde{O}}_{2}\left(X,Y\right)\;.$$

The operator ${\mathrm{Tr}}^{\prime }$ found in Equations (17) and (18) takes the trace over the quantum operators $\hat{x}$, while ${\tilde{O}}_{1}$ and ${\tilde{O}}_{2}$ are two arbitrary mixed Weyl symbols.

### Constant Temperature Quantum–Classical Dynamics

In order to illustrate the advanced techniques for controlling the temperature of the harmonic modes, we consider a simple system with just two phononic modes, with coordinates $\left(X_{1},X_{2}\right)$, and two NHC chains of length one (which is usually enough to generate ergodic dynamics for stiff harmonic degrees of freedom) [93,94,95]. Thus, the extended phase space point can be written as $X^{e}$= $(R_{1}$,$\eta_{1}^{\left(1\right)},$$\eta_{2}^{\left(1\right)},$$R_{2},$ $\eta_{2}^{\left(1\right)},$$\eta_{2}^{\left(2\right)},$$P_{1},$$P_{\eta_{1}}^{\left(1\right)},$ $P_{\eta_{1}}^{\left(2\right)},$$P_{2},$$P_{\eta_{2}}^{\left(1\right)},$$P_{\eta_{2}}^{\left(2\right)});$ consequently, the extended phase space gradient is $\nabla^{e}$= $(\left(\partial /\partial R_{1}\right),$ $(\partial /\partial \eta_{1}^{\left(1\right)}),$ $(\partial /\partial \eta_{2}^{\left(1\right)}),$ $(\partial /\partial R_{2}),$ $(\partial /\partial \eta_{1}^{\left(2\right)}),$ $\left(\partial /\partial \eta_{2}^{\left(2\right)}\right)$, $(\partial /\partial P_{1}),$ $(\partial /\partial P_{\eta_{1}}^{\left(1\right)}),$ $\left(\partial /\partial P_{\eta_{2}}^{\left(1\right)}\right)$, $(\partial /\partial P_{2}),$ $\left(\partial /\partial P_{\eta_{1}}^{\left(2\right)}\right)$, $(\partial /\partial P_{\eta_{2}}^{\left(2\right)}).$

If we now define the antisymmetric matrix $R=-R^{-1}$ as

(19) $$\begin{array}{c} R=\left[\begin{array}{cccccccccccc} 0 & 0 & 0 & 0 & 0 & 0 & 1 & 0 & 0 & 0 & 0 & 0\\ 0 & 0 & 0 & 0 & 0 & 0 & 0 & 1 & 0 & 0 & 0 & 0\\ 0 & 0 & 0 & 0 & 0 & 0 & 0 & 0 & 1 & 0 & 0 & 0\\ 0 & 0 & 0 & 0 & 0 & 0 & 0 & 0 & 0 & 1 & 0 & 0\\ 0 & 0 & 0 & 0 & 0 & 0 & 0 & 0 & 0 & 0 & 1 & 0\\ 0 & 0 & 0 & 0 & 0 & 0 & 0 & 0 & 0 & 0 & 0 & 1\\ -1 & 0 & 0 & 0 & 0 & 0 & 0 & -P_{1} & 0 & 0 & 0 & 0\\ 0 & -1 & 0 & 0 & 0 & 0 & P_{1} & 0 & -P_{M_{\eta_{2}}}^{\left(1\right)} & 0 & 0 & 0\\ 0 & 0 & -1 & 0 & 0 & 0 & 0 & P_{M_{\eta_{2}}}^{\left(1\right)} & 0 & 0 & 0 & 0\\ 0 & 0 & 0 & -1 & 0 & 0 & 0 & 0 & 0 & 0 & -P_{2} & 0\\ 0 & 0 & 0 & 0 & -1 & 0 & 0 & 0 & 0 & P_{2} & 0 & -P_{\eta_{1}}^{\left(2\right)}\\ 0 & 0 & 0 & 0 & 0 & -1 & 0 & 0 & 0 & 0 & P_{\eta_{1}}^{\left(2\right)} & 0\;. \end{array}\right] \end{array}$$

together with the mixed Weyl symbol of the extended Hamiltonian

(20) $$\begin{array}{ccc} {\tilde{H}}^{e}\left(X^{e}\right) & = & {\hat{H}}_{S}+H_{B}\left(X\right)+{\tilde{V}}_{\mathrm{SB}}\left(R\right)+\sum_{I=1}^{2}\sum_{L=1}^{2}\frac{P_{\eta_{L}}^{\left(I\right)}}{2M_{\eta_{L}}}+\sum_{I=1}^{2}\sum_{L=1}^{2}k_{B}T^{\left(I\right)}\eta_{L}^{\left(I\right)}\;. \end{array}$$

the QC equation of motion at constant temperature can be written in compact form [79,80,81] as

(21) $$\begin{array}{ccc} \partial_{t}{\tilde{O}}^{e}\left(t\right) & = & \frac{i}{\hbar }\left[\begin{array}{cc} {\tilde{H}}^{e} & {\tilde{O}}^{e}\left(t\right) \end{array}\right]\Omega \left[\begin{array}{c} {\tilde{H}}^{e}\\ {\tilde{O}}^{e}\left(t\right) \end{array}\right]-\frac{1}{2}{\tilde{H}}^{e}\overset{\leftarrow }{\nabla^{e}}R\vec{\nabla^{e}}{\tilde{O}}^{e}\left(t\right)\\ & + & \frac{1}{2}{\tilde{O}}^{e}\left(t\right)\overset{\leftarrow }{\nabla^{e}}R\vec{\nabla^{e}}{\tilde{H}}^{e}\;, \end{array}$$

where ${\tilde{O}}^{e}\left(t\right)={\tilde{O}}^{e}\left(X^{e},t\right)$; the NHC variables are $\left(\eta_{L}^{\left(I\right)},P_{\eta_{L}}^{\left(I\right)}\right)$, with *I* and *L* running over the phonons and the coordinates of the chain, respectively; $k_{B}$ is the Boltzmann constant; $T^{\left(I\right)}$ is the temperature of each mode; and $M_{\eta_{L}^{\left(I\right)}}$ are the inertial parameters of the NHC variables.

Constant temperature averages and correlation functions can be calculated by choosing the mixed Weyl symbol ${\tilde{W}}^{e}\left(X^{e}\right)$ of the statistical operator in extended space as

(22) $$\begin{array}{ccc} {\tilde{W}}^{e}\left(X^{e}\right) & = & {\hat{w}}_{S}W^{\beta }\left(X\right)\prod_{I=1}^{2}\prod_{L=1}^{2}\delta \left(\eta_{L}^{\left(I\right)}\right)\delta \left(P_{\eta_{L}}^{\left(I\right)}\right)\; \end{array}$$

where ${\hat{w}}_{S}$ is the mixed Weyl symbol of the statistical operator of the quantum subsystem while the thermal mixed Weyl symbol of the statistical operators of the phonons is

(23) $$\begin{array}{ccc} W^{\beta }\left(X\right) & = & \prod_{I=1}^{2}\frac{\tanh(\beta \omega_{I}/2)}{2}\exp\left[-\frac{2\tanh(\beta \omega_{I}/2)}{\omega_{I}}\left(\frac{P_{I}^{2}}{2}+\frac{\omega_{I}^{2}}{2}R_{I}^{2}\right)\right]\;, \end{array}$$

where $\beta =1/k_{B}T$ and $\omega_{I}$ is the frequency of phonon *I*. If, in the mixed Weyl symbol of the Hamiltonian in Equation (20), one defines $T^{\left(I\right)}=T\;\forall I$, then the dynamics defined by Equation (21) defines constant-temperature evolution. Instead, the choice of $T^{\left(I\right)}=1/k_{B}\beta^{\left(I\right)}$ with

(24) $$\beta^{\left(I\right)}=\frac{2\tanh(\beta \omega_{I}/2)}{\omega_{I}}\;,\;\forall I\;.$$

describes a time evolution of the phonons, where zero-point effects are taken into account. The structure of the extended QC super-propagator ${\tilde{\tilde{U}}}^{e}$ is similar to that displayed in Equation (15):

(25) $$\begin{array}{ccc} {\tilde{\tilde{U}}}^{e}\left(t\right) & = & \exp\left\{\left(it/\hbar \right)\left[\begin{array}{cc} {\tilde{H}}^{e} & \ldots \end{array}\right]\Omega \left[\begin{array}{c} {\tilde{H}}^{e}\\ \ldots \end{array}\right]-\left(t/2\right)\left({\tilde{H}}^{e}{\overset{\leftarrow }{\nabla }}^{e}R{\vec{\nabla }}^{e}\ldots \right)\right.\\ & + & \left.\left(t/2\right)\left(\ldots {\overset{\leftarrow }{\nabla }}^{e}R{\vec{\nabla }}^{e}{\tilde{H}}^{e}\right)\right\}\;. \end{array}$$

Since we are interested in thermal and zero-point QC averages and correlation functions of non-fictitious dynamical variables, we must consider mixed Weyl symbols $\tilde{O}\left(X\right)$ that, at t=0, do not depend on the extended phase space point $X^{e}$ but depend on the non-fictitious phase space point *X*. However, the key to temperature control is that the phase space variable dependence found at t=0 is not preserved at t≠0. We have ${\tilde{\tilde{U}}}^{e}\left(t\right)\tilde{O}\left(X\right)=\tilde{O}\left(X^{e},t\right)$. Finally, we can write the expression for thermal (or zero-point) QC averages as

(26) $$\begin{array}{ccc} {\langle \tilde{O}\left(X,t\right)\rangle }_{e} & = & {\mathrm{Tr}}^{\prime }\int dX^{e}\;W^{e}\left(X^{e}\right)\tilde{O}\left(X,t\right)\;, \end{array}$$

(27) $$\begin{array}{ccc} {\langle {\tilde{O}}_{1}\left(X,t\right){\tilde{O}}_{2}\left(X\right)\rangle }_{e} & = & {\mathrm{Tr}}^{\prime }\int dX^{e}\;W^{e}\left(X^{e}\right){\tilde{O}}_{1}\left(X,t\right){\tilde{O}}_{2}\left(X\right)\;. \end{array}$$

## 8. Conclusions

In this work, we brought to light a parallel between the bipartite structure of human logic and the quantum–classical view of physical phenomena. We discussed that one finds both Aristotelian logic and non-Aristotelian logic in the human psyche. Aristotelian logic explains the behavior of the classical world, whereas non-Aristotelian logic applies to the quantum world. We would like to remind the reader that, in the manuscript, the word ‘psyche’ means the set of collective brain phenomena emerging from microscopic cells’ dynamics, and is not anything metaphysical.

We have been motivated by the analogy with bi-partite logic to propose a quantum–classical model for studying brain processes. One idea behind this proposal, i.e., the need to mix a quantum and classical level of description together, had already been supported in a somewhat less explicit form by three theoretical approaches, which we reviewed in the first part of this work. Two approaches that we reviewed were originally designed by their authors as theories of consciousness. However, this is not the perspective from which we looked at them. In this paper, we were not interested in describing consciousness. Instead, we interpreted these theories in terms of purely physical processes, and it was only in such a respect that we considered them.

The very formulation of our model is given in terms of quantum and classical variables that are treated on the same level. It does not need to invoke, e.g., quantum gravitational effects in the brain. Instead, the crux is that the quantum variables play the fundamental role of providing a quantum guiding mechanism for the classical variables that they are coupled with. Such an idea was originally formulated by Pascual Jordan. With respect to this, in order to have quantum effects in brain dynamics, there is no need to invoke a highly improbable coherent quantum state of the whole brain at a high temperature. There are important quantum properties of few-body systems that are not lost at a high temperature. These were discussed in the text. What is needed for the quantum biology of the brain was again suggested long ago by Pascual Jordan: The collapse of the wave function works as an amplification mechanism acting as a bridge between the quantum and the classical world.

We did not perform actual numerical calculation but we introduced a general quantum–classical Hamiltonian model, which can be specialized to describe quantum particles and spins interacting with various types of environments, such as those found in neurons and astrocytes, or at the sub-neuronal level in, e.g., tubulins. Moreover, electromagnetic DOFs can also be described by our model Hamiltonian. We showed that the quantum–classical theory provides a statistical mechanic formulation of averages and correlation functions. In turn, as is well-known, correlation functions lead to the definition of response functions. Non-invasive brain stimulation techniques can provide the numerical data to which our theory can be compared.

We took the risk to discuss many complex ideas using only logic and our scientific knowledge. We presented a synthesis of subtle concepts, introduced our quantum–classical model, with which we plan to take on big scientific challenges, and declared the direction that our future work will take. We carried this out with the belief that science is not only made by numbers, but also made by understanding and sharing concepts with the community. Subsequently, such concepts can be discussed and refined, possibly leading to new advancements. Our future work will be devoted to interpreting biochemical processes in the brain in terms of quantum–classical dynamics. This will also require performing quantum–classical calculations of neural response functions. The implications of the interplay between the bipartite structures of both the world and the psyche will be investigated through the formulation of quantum–classical models of decision making.

## Data Availability

Not applicable.

## Abbreviations

The following abbreviations are used in this manuscript:

ACS	alternating current stimulation
CaMKII	calcium-calmodulin kinase II
DOF	degrees of freedom
DQMB	dissipative quantum model of brain
EMF	electromagnetic field
GS	general semantics
MAPs	microtubule-associated proteins
NHC	Nosé–Hoover chain
Orch OR	orchestrated objective reduction
QC	quantum–classical
QFT	quantum field theory
QFTMB	quantum field theory model of brain
SSB	spontaneous symmetry breaking
tDCS	transcranial direct-current stimulation
